# The Profiles and Correlates of Psychopathology in Adolescents and Adults with Williams, Fragile X and Prader–Willi Syndromes

**DOI:** 10.1007/s10803-019-04317-1

**Published:** 2019-12-04

**Authors:** R. Royston, C. Oliver, P. Howlin, A. Dosse, P. Armitage, J. Moss, J. Waite

**Affiliations:** 1 The Cerebra Centre for Neurodevelopmental Disorders, School of Psychology, University of Birmingham, Edgbaston, Birmingham, B15 2TT UK; 2 Department of Psychology, King’s College London, Strand, London, WC2R 2LS UK; 3 Institute of Cognitive Neuroscience, University College London, Alexandra House, 17 Queen Square, London, WC1N 3AZ UK; 4 Present Address: School of Life & Health Sciences, Aston University, Birmingham, B4 7ET UK; 5 Present Address: Division of Psychiatry, University College London, London, W1T 7NF UK

**Keywords:** Williams syndrome, Prader–Willi syndrome, Fragile X syndrome, Psychopathology, Correlates

## Abstract

Psychopathology is prevalent in Williams (WS), fragile X (FXS) and Prader–Willi (PWS) syndromes. However, little is known about the potential correlates of psychopathology in these groups. A questionnaire study was completed by 111 caregivers of individuals with WS (n = 35); FXS (n = 50) and PWS (n = 26). Mean age was 26 years (range 12–57 years); 74 (67%) were male. Multiple regression analyses indicated that higher rates of health problems and sensory impairments predicted higher psychopathology in WS (p < .0001). In PWS, poorer adaptive ability predicted higher overall psychiatric disturbance (p = .001), generalised anxiety (p = .006) and hyperactivity (p = .003). There were no significant predictors in FXS. This study highlights dissociations in the potential risk markers of psychopathology between genetic syndromes. Implications for intervention are discussed.

**Electronic supplementary material:**

The online version of this article (10.1007/s10803-019-04317-1) contains supplementary material, which is available to authorized users.

The prevalence of psychopathology in individuals with intellectual disabilities (ID) is estimated as being four to five times higher than in the general population (Matson and Shoemaker 2011). A number of syndromes associated with ID evidence differing profiles of psychopathology. For example, individuals with Prader–Willi syndrome are reported to have a high prevalence of psychotic illness (Soni et al. 2008), whilst social anxiety is associated with fragile X and Turners syndromes (Cordeiro et al. 2011; Lesniak-Karpiak et al. 2003). Generalised anxiety and phobias are prominent in Williams syndrome (Dodd and Porter 2009; Leyfer et al. 2006; Royston et al. 2017) and separation anxiety is common in Cornelia de Lange syndrome (Crawford et al. 2017). Study of psychopathological profiles and correlates may help to inform causal models of psychiatric disturbance in individuals with ID. Cross syndrome comparisons also facilitate exploration of within-syndrome psychopathological risk, as well as identifying commonalities between genetic syndromes.

In this study we examine the nature and correlates of psychopathology in three syndromes that are broadly comparable for degree of ID: Williams (WS), fragile X (FXS) and Prader–Willi syndromes (PWS). WS is caused by a microdeletion of approximately 28 genes on chromosome 7q11.23, with an estimated prevalence of up to 1 in 7500 births (Ewart et al. 1993; Strømme et al. 2002). The WS phenotype is characterised by hyper-sociability, hypersensitivity to sound (hyperacusis), attention deficits, impulsivity and emotional difficulties (Einfeld et al. 1997; Jones et al. 2000; Levitin et al. 2005; Leyfer et al. 2006). FXS is caused by a fMR1 gene mutation at Xq27.3, with a prevalence of 1 in 5000 men and 1 in 4000–6000 women (Coffee et al. 2009; Saldarriaga et al. 2014). Repetitive behaviour, attentional difficulties, social and sensory impairments and mental health problems such as anxiety are common (Garber et al. 2008; McLennan et al. 2011; Oakes et al. 2016; Rogers et al. 2003). PWS results from the absence of the expression of the paternal gene on chromosome 15q11.2–q13, affecting 1 in 15,000–30,000 individuals (Cassidy and Driscoll 2009; Cassidy et al. 2012). This syndrome is associated with obsessions, compulsions, temper outbursts and anxiety (Cassidy et al. 2011; Dykens and Kasari 1997; Reddy and Pfeiffer 2007). Further detailed descriptions of these syndromes can be found in reviews by Cassidy and Driscoll (2009); Mazzocco (2000) and Royston et al. (2019).

In each of these syndromes there is a heightened risk of developing psychopathology although the types of disorder differ between the groups. Thus, the prevalence of generalised anxiety disorder is high in WS and FXS but not PWS; rates of social anxiety disorder are elevated in FXS but not WS or PWS and there is a higher prevalence of obsessive–compulsive disorder in PWS but not FXS (Cordeiro et al. 2011; Dykens and Shah 2003; Leyfer et al. 2006; Royston et al. 2017). However, the profiles of psychopathology in these syndromes and their correlates have not been compared directly using the same assessments.

Both within and between syndromes, identification of correlates or risk markers could provide information on putative causal pathways of psychopathology and may have implications for early intervention. There has been little investigation of potential correlates of psychopathology in WS, FXS or PWS and among the few variables that have been explored, there seems to be no relationship with gender (Cordeiro et al. 2011; Einfeld et al. 1999; Dykens and Kasari 1997; Riby et al. 2014; Stinton et al. 2010), and data on the association with age and intellectual functioning are inconsistent (Cordeiro et al. 2011; Dodd and Porter 2009; Dykens 2004; Dykens and Cassidy 1995; Dykens and Kasari 1997; Hessl et al. 2001; Lesniak-Karpiak et al. 2003; Stinton et al. 2010). However, in each of these syndromes there is a raised prevalence of physical health problems; significant difficulties in sensory processing are also typical (Levitin et al. 2005; Rogers et al. 2003; Stauder et al. 2002). Auditory sensory sensitivities, in particular, are prevalent in all groups (Ethridge et al. 2016; Levitin et al. 2005; Stauder et al. 2002), and in WS, for example, there is evidence that that many specific phobias are related to hypersensitivity to noise (Leyfer et al. 2006; Royston et al. 2017). Nevertheless, there has been no research into the impact of these characteristics, despite both poor physical health and sensory processing difficulties being potentially influential in the development of anxiety, depression and other disorders (Pober and Morris 2007; Uljarević et al. 2018).

Discrepancies in reported rates of psychopathology across syndromes, and variability in the reported correlations with other factors may reflect differences in assessment methodologies and categorisation systems. Typically, psychopathology in individuals with ID is examined using assessments based on diagnostic taxonomies developed for the general population and judgments about the presence or absence of a disorder are based on predefined symptoms and criteria (American Psychiatric Association 2013). However, the applicability of this approach for individuals with ID is uncertain, due to possible differences in clinical features and behaviours compared with individuals in the general population. In recent years, growing recognition that disorders may exist along a continuum from normality to abnormality (Sanislow et al. 2010) has resulted in a move towards the use of dimensional frameworks to assess psychopathology. This approach allows for specification of disorder severity and is more resistant to minor fluctuations in psychopathological difficulties (Clark et al. 1995; Watson 2005). Dimensional assessments are also often developed using empirical literature and psychometric research to form assessment subscales and categories that are not pre-defined and may be specific to certain groups, and thus are potentially more relevant to psychopathology in individuals with ID.

In this study, we used both categorical and dimensional questionnaire measures to explore phenomenology and cross-syndrome differences in individuals with WS, FXS and PWS. We also investigated associations between psychopathology and age, adaptive ability (as a proxy measure of intellectual functioning), health problems and auditory sensory processing. Our focus was specifically on adolescents and adults because of the high rates of mental health problems in these age groups.

The aims of the study were:

1. To describe the profile of psychopathology in individuals with WS, FXS and PWS and identify differences in profiles between the syndromes.

We hypothesised that social anxiety would be significantly higher in FXS; generalised anxiety would be higher in FXS and WS compared to PWS; obsessive–compulsive behaviour would be higher in PWS.

2) To assess whether age, adaptive ability, health difficulties and auditory sensory processing impairments are associated with psychological disturbance within syndromes.

We hypothesised that health difficulties and auditory sensory processing impairments would be positively associated with psychopathology for all groups.

## Methods

### Participants

Participants were recruited from the Cerebra Centre for Neurodevelopmental Disorders (CCND) participant database (response rate: 38% for PWS, 18% for FXS and 36% for WS). Participants were also recruited from the Fragile X Society, the Williams Syndrome Foundation in the UK, the Canadian Association for Williams Syndrome and the Williams Syndrome Registry (www.williams-syndrome.org/registry), administrated by the Williams Syndrome Association, USA.

A total of 132 families participated in the survey. Participants were included if they had completed at least 25% of the questionnaire survey (10 individuals excluded) and the person they cared for was over the age of 12 (2 excluded). Inclusion criteria were a confirmed genetic diagnosis from a clinician (clinical geneticist, paediatrician or general practitioner) or a positive result from prior genetic testing. Individuals with mobility difficulties (n = 3) and limited verbal ability (speaks/signs fewer than 30 words, n = 5) were excluded to ensure better group matching. Due to the tendency for higher cognitive functioning in females with FXS (Bartholomay et al. 2019), only males with FXS were included in the study. This resulted in a total of 110 participants (mean age = 26.53, *SD *= 10.36, range:12–57, 74 male). There were 35 participants in the WS sample (mean age = 25.51, *SD *= 12.39, range: 12–57, 14 male), 49 in the FXS sample (mean age = 27.08, *SD *= 9.18, range: 12–50, all male) and 26 in the PWS group (mean age = 26.85, *SD *= 9.77, range: 12–47, 11 male).

This study was conducted as part of a larger study investigating behavioural phenotypes in neurodevelopmental disorders. The study was approved by the NHS Coventry and Warwickshire REC committee as an amendment to an existing ethics application (reference: 10/H1210/01).

### Measures

#### Waisman Activities of Daily Living Scale

The Waisman Activities of Daily Living Scale **(**W-ADL; Maenner et al. 2013) is designed for adolescents and adults (over the age of 10) with ID and examines ability level and independence in carrying out daily living activities. This measure of adaptive skills can be used as a proxy measure of functional ability. The informant scale has 17 items, each rated on a three-point Likert scale; 2 = ‘Independent or does on own’, 1 = ‘does with help’ and 0 = ‘does not do at all’. The scale is reported to have high internal consistency in a sample of adolescents and adults aged 10–52 years (Cronbach’s alphas ranging from .88–.94).

#### Health Questionnaire

The Health questionnaire (Hall et al. 2008) examines the presence and severity of current (in the last month) and lifetime health problems in children and adults. The questionnaire consists of 15 health problems scored on a scale of 0 = “never affected” to 3 = “severely affected”. Scores are summed to obtain an overall health score. Item level reliability was .72 for lifetime problems and .76 for current problems (Hall et al. 2008).

#### Developmental Behaviour Checklist

The Developmental Behaviour Checklist (DBC; Einfeld and Tonge 1995) measures emotional and behavioural disorders in individuals with intellectual disabilities over the past six months. Behaviour is scored as 0 = ‘not true as far as you know’, 1 = ‘somewhat or sometimes true’ or 2 = ‘very true or often true’. The DBC-P is the parent version for individuals under the age of 18 and comprises 95 items and five subscales; disruptive/antisocial, self-absorbed, communication, anxiety and social relating. It has good test–retest (.83) and inter-rater reliability (Einfeld and Tonge 1995). The DBC-A is the adult version suitable for individuals over the age of 18. This version contains 107 items and six subscales; antisocial, self-absorbed, communication and anxiety disturbance, disruptive, social relating and depressive. The DBC-A has high internal consistency (α = .95) and high test–retest reliability at two weeks for both paid carers and family carers at .75 and .85 respectively (Mohr et al. 2005).

The DBC-P and DBC-A are not directly comparable due to differing subscales. However, mean item scores, positively checked items and the intensity score of each measure can be calculated and combined (Taffe et al. 2008). This study examines the subscales for group differences but mainly focuses on the mean item score (sum of items divided by the number of items) and the intensity score (proportion of items scored as ‘very true of often true’). Whilst the DBC is generally regarded as categorical, the mean scores provide a dimensional measure of assessment.

#### Anxiety, Depression and Mood Scale

The Anxiety, Depression and Mood Scale (ADAMS; Esbensen et al. 2003) is an informant questionnaire comprising 28 items on four-point rating scales, with five subscales; manic/hyperactive behaviour, depressed mood, social avoidance, general anxiety and compulsive behaviour. The questionnaire has been validated in individuals with ID aged 10–79 years and has good test–retest reliability (.81). Internal consistency for the subscales and total score were calculated for this study and coefficients were high (manic hyperactive behaviour = .88, depressed mood = .88, social avoidance = .85, general anxiety = .87, compulsive behaviour = .83, total score = .93).

In this study, the ADAMS subscales were highly correlated with each other for every syndrome group, indicating high interrelatedness between categorical constructs (Online Resource A). Correlations between the ADAMS subscales and the DBC mean item and intensity score were also assessed. Overall, the measures were highly correlated, with the ADAMS total score significantly correlating with DBC mean item score (r_s_ = .80, p < .0001) and DBC intensity score (r_s_ = .67, p < .0001) (full table of correlations available in Online Resource B).

#### Sensory Experiences Questionnaire

The Sensory Experiences Questionnaire (SEQ; Baranek et al. 2006) is an informant questionnaire assessing sensory processing in daily tasks for children with autism and developmental delays. The items reflect five sensory domains: Gustatory-Olfactory, Tactile, Auditory, Visual and Vestibular-Proprioceptive. Caregivers are asked to report the frequency of sensory behaviours on a five-point scale; ‘almost never’ to ‘almost always’. The SEQ has excellent internal consistency, Cronbach’s alpha = .80 and test–retest reliability, ICC = .92 (Little et al. 2011). This questionnaire has been utilised in samples including both children and adults with neurodevelopmental disorders (e.g, Kolacz et al. 2018). For the purpose of this study, only the auditory section of the SEQ was administered as this was the main domain of interest.

#### Procedure

Parents/carers were sent a link via email to online information sheets and consent forms. Where consent was provided, the link directed individuals to the online survey study, consisting of all the questionnaire measures. Participants aged 16 or over with the capacity to consent were asked to complete a paper consent form or provide consent through an online website link. The decision as to whether an individual was able to give consent was taken by the parent/carer and written information to provide guidance on how to assess capacity was provided by researchers.

Parents/carers of individuals aged 16 or over without the capacity to consent for themselves and parents/carers of individuals under 16 years old provided assent online or on paper. Breaks in the survey were included and paper copies were available on request. All participants taking part in the survey received an individualised feedback report.

#### Data Analysis

**Fig. 1:**
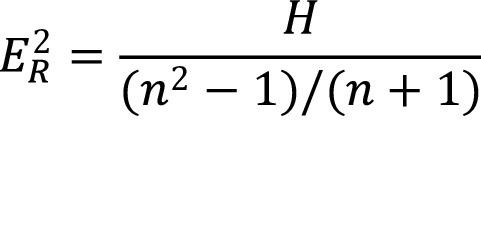
Epsilon squared formula for Kruskal–Wallis tests as recommended by Tomczak and Tomczak (2014)

Missing data were prorated according to the assessment manuals or authors’ instructions. The sample collected for the DBC-P was too small to conduct statistical analyses, therefore only the DBC-A subscales or the combined DBC mean item score and intensity scores are presented in the analyses. All data were tested for normality using Shapiro Wilks tests. The use of parametric and non-parametric tests was chosen on a case by case basis, although the majority of the data was not normally distributed. Kruskal–Wallis tests with Dunn’s (1964) procedure post hoc tests, Chi square tests and one-way ANOVAs with Gabriel’s pairwise test procedure were utilised to assess group differences. Due to the large number of analyses, only group differences are presented. Partial eta squared (η_p_^2^) and epsilon squared (ε^2^; see Fig. 1) were calculated to estimate effect sizes.

Interpretation of effect sizes was based on Cohen’s guidelines; small = .2, medium = .5, large = .8 (Cohen 1988). Multiple hierarchical regression analyses were then conducted to identify predictors of psychopathology. A hierarchical regression was chosen to allow for the inputting of the potential confounders (age and adaptive ability) in the first step of the analysis. This decision was based on the associations of these variables with other associated factors identified in the general population (Humes et al. 2013; Li and Lindenberger 2002). The inclusion of health and sensory processing in the second step enabled the examination of the additional contribution of these variables in the prediction of psychopathology.

The majority of the data was normally distributed although some variables violated the assumption of homoscedasticity. To overcome this, bootstrapping with bias corrected and accelerated 95% confidence intervals were used for all analyses. The data were assessed for outliers using Cook’s distance, casewise diagnostics, leverage and student deleted residuals. Data with high leverage were retained in the analyses, as it is suggested that this does not exert a large influence on regression coefficients (Field 2013). Individual analyses were rerun excluding outliers and the results presented are without outliers as this did not significantly change results.

Assumption testing for the regression analyses are presented in Table 1. Durbin-Watson tests identified little autocorrelation for each group. Multicollinearity was tested using variation inflation factors, which identified acceptable levels of correlation between predictors. The predictor ‘age’ violated the assumption of linearity for the WS group and data were transformed using inverse transformation for all groups.

**Table 1:** Autocorrelation and multicollinearity assumption testing for regression analyses

	Durbin Watson mean (range)	Variation inflation factors (range)
Williams syndrome	2.07 (1.84–2.33)	1.09–1.96
Fragile X syndrome	2.21 (1.65–2.68)	1.09–1.49
Prader–Willi syndrome	2.16 (1.48–2.74)	1.24–2.93

A *p* value of .01 was chosen for the analyses to adjust for multiple comparisons. The use of a Bonferroni correction was viewed as too stringent and due to the exploratory nature of genetic syndrome research, it was considered of higher importance to prevent Type II errors and to identify possible relationships which could then be addressed in future research.

## Results

### Group Differences

Prior to other analyses, group differences in auditory sensory processing scores, health, age, gender, adaptive ability and psychopathology were examined(Table 2). There were no differences between the groups for age or adaptive ability, although there was an expected gender difference χ^2^(2) = 43.02, *p *= <.0001, Cramer’s V = .63, with the FXS group including more males (100%) than the WS (40%) and PWS (42%) groups. Auditory sensory processing scores were significantly different between the groups (F(2107) = 19.67, η_p_^2^ = .27, *p *< .0001). Pairwise comparisons revealed that individuals with WS (mean = 18.69) and FXS (17.69) had higher auditory processing impairments than individuals with PWS (13.19). Group differences were also found for overall lifetime health (χ^2^(2) = 23.90, ε^2^ = .22, *p *< .0001), with WS (74.74) scoring higher for health difficulties compared with FXS (40.52). For overall current health problems, significantly more health problems were found in WS (66.80) and PWS (62.85) compared with FXS (42.15) (χ^2^(2) = 15.15, ε^2^ = .14, *p *= .001).

**Table 2:** Group differences for psychopathology and other variables between individuals with Williams syndrome, fragile X syndrome and Prader–Willi syndrome

	Domain		WS	FXS	PWS	Post-hoc
Gender	Males	%	40	100	42	FXS > PWS, WS
SEQ	Auditory score	m (SD) range	18.7 (3.4) 13–25	17.6 (3.9) 10–26	13.2 (3.1) 8–20	FXS, WS > PWS
Health	Lifetime overall scores	med (IR) range	9.0 (4.0) 2–22	5.0 (3.0) 0–16	6.0 (8.0) 1–23	WS > FXS
	Current overall scores		3.0 (5.0) 0–12	0.0 (2.0) 0–16	2.0 (3.3) 0–19	PWS, WS > FXS
ADAMS	General anxiety		7.0 (6.0) 0–21	7.5 (7.0) 0–20	4.0 (8.0) 0–12	FXS, WS > PWS
	Manic hyperactive		4.0 (7.0) 0–12	7.0 (7.0) 0–14	3.0 (6.5) 0–12	FXS > PWS
	Social avoidance		3.0 (5.0) 0–16	8.0 (7.0) 2–17	2.0 (5.3) 0–12	FXS > PWS, WS
DBC A	Social relating		3.0 (4.0) 0–13	8.0 (4.8) 3–14	4.0 (4.0) 0–12	FXS > PWS, WS

*m* mean, *SD* standard deviation, *med* median, *IR* interquartile range

To examine the profile of psychopathology in WS, FXS and PWS, group differences were assessed using the ADAMS and DBC assessment measures. Based on the ADAMS, general anxiety was the highest scoring domain for individuals with WS (median = 7.0, IR = 6.0, range = 0–21), social avoidance for individuals with FXS (median = 8.0, IR = 7.0, range = 2–17) and depressed mood for those with PWS (median = 5.0, IR = 7.0, range = 0–18) (for a full descriptive summary, see Online Resource C). Table 2 displays the group differences for the measures of psychopathology and the individual variables. There were group differences on the ADAMS for: 1) general anxiety (χ^2^(2) = 9.61, ε^2^ = .09, *p *= .008) with FXS (60.29) and WS (61.34) scoring higher than PWS (38.62), 2) manic/hyperactive (χ^2^(2) = 10.16, ε^2^ = .09, *p *= .006) with FXS (65.87) scoring higher than PWS (43.02), and 3) social avoidance (χ^2^(2) = 37.13, ε^2^ = .34, *p *< .0001) with FXS (76.09) scoring higher than WS (39.17) and PWS (38.67). On the DBC-A, only the social relating subscale differed between groups (χ^2^(2) = 26.96, ε^2^ = .32, *p *< .0001). Individuals with FXS (58.14) scored higher for social relating difficulties than individuals with PWS (34.93) and WS (27.28). There were no other differences between the groups on either measure.

### Correlates of Psychopathology

To assess whether the full model of individual variables considered together was associated with psychological disturbance within syndromes, hierarchical regressions were conducted. The ANOVA models for the hierarchical regressions are presented in Table 3 (full models including β coefficients and confidence intervals for individual predictors are available in Online Resource D). Step 1 of the regression model included age and adaptive functioning as predictors, and Step 2 included all factors; age, adaptive functioning, auditory sensory processing and health problems. The outcome variables included all the ADAMS subscales (depressed mood, general anxiety, manic/hyperactive, obsessive compulsive, social avoidance) and the total score, and the DBC mean and intensity scores.

**Table 3:** Overall ANOVA models and R^2^ for each scale of psychopathology for Williams syndrome, Fragile X syndrome and Prader–Willi syndrome

	Total score	Depressed mood	Generalised anxiety	Manic hyperactivity	Obsessive compulsive	Social avoidance	DBC mean item score	DBC intensity
Williams syndrome								
Step 1								
F statistic	1.30	1.14	.91	1.63	.73	1.29	2.91	2.50
*df*	(2,32)	(2,31)	(2,32)	(2,32)	(2,32)	(2,31)	(2,31)	(2,31)
R^2^	.08	.07	.05	.09	.04	.08	.16	.14
Step 2								
F statistic	6.25***	5.67*	4.77*	3.92*	2.20	7.12***	8.16***	5.92*
*df*	(5,29)	(5,28)	(5,29)	(5,29)	(5,29)	(5,28)	(5,28)	(5,28)
R^2^	.52	.50	.45	.40	.28	.56	.59	.51
Fragile X syndrome								
Step 1								
F statistic	.35	.14	.07	1.80	1.26	1.28	4.86	2.38
*df*	(2,45)	(2,43)	(2,45)	(2,45)	(2,45)	(2,45)	(2,44)	(2,43)
R^2^	.02	.01	.00	.07	.05	.05	.18	.10
Step 2								
F statistic	1.35	1.43	1.34	1.45	2.29	.74	5.01*	2.49
*df*	(5,42)	(5,40)	(5,42)	(5,42)	(5,42)	(5,42)	(5,41)	(5,40)
R^2^	.14	.15	.14	.15	.21	.08	.38	.24
Prader–Willi syndrome								
Step 1								
F statistic	3.97	4.00	3.37	5.31	1.41	2.08	2.70	2.19
df	(2,23)	(2,22)	(2,23)	(2,23)	(2,23)	(2,23)	(2,23)	(2,23)
R^2^	.26	.27	.19	.32	.11	.15	.19	.09
Step 2								
F statistic	2.71	3.53	2.51	3.62	1.49	1.40	3.49	2.33
*df*	(5,20)	(5,19)	(5,20)	(5,20)	(5,20)	(5,20)	(5,20)	(5,20)
R^2^	.40	.48	.35	.48	.27	.26	.47	.37

*p < .01; **p < .001; ***p < .0001

### Williams Syndrome

In WS, the overall model of predictors was significant for every outcome variable, except for the obsessive–compulsive subscale (F(5,29) = 2.20, p = .081, R^2^ = .28). For all other subscales, age and adaptive ability in Step 1 did not produce significant results, though the addition of health and sensory processing to the model in Step 2 led to statistically significant increases in the predictive ability of the model, which accounted for an additional 31–48% of the variance.

**Fig. 2:**
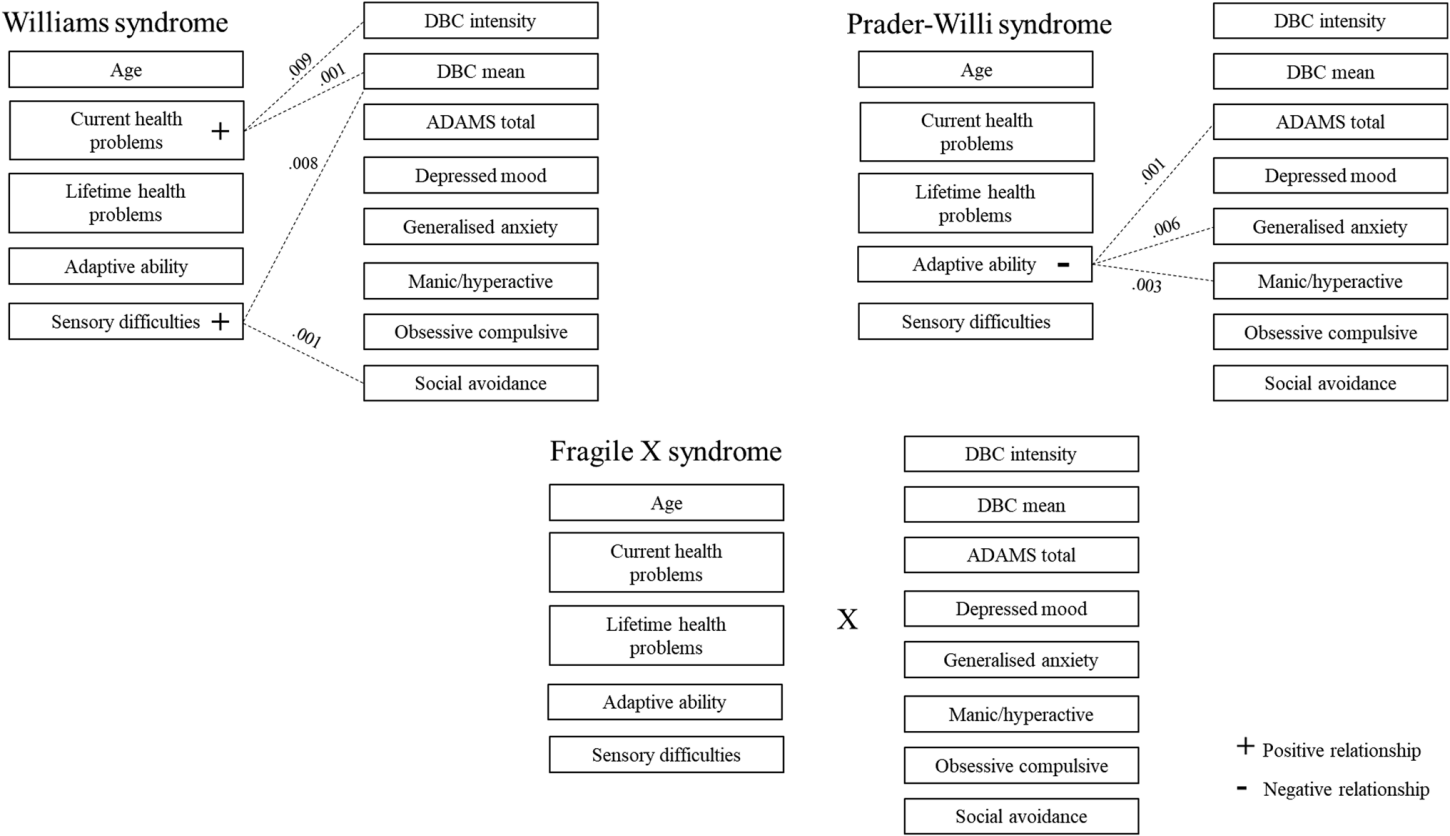
Predictors of psychopathology for Williams syndrome, Prader–Willi syndrome and Fragile X syndrome

The influence of the individual predictors (age, adaptive ability, health difficulties and auditory sensory processing impairments) on the psychopathology scales is illustrated in Fig. 2. In WS, more current health problems were predictive of higher DBC mean item score (b = .05 [95% CI: .02, .09], β = .52, p = .001) and DBC intensity (b = .02 [95% CI: .01, .04], β = .55, p = .009). Higher auditory sensory difficulties significantly predicted a higher DBC mean (b = .04 [95% CI: .01, .06], β = .50, p = .008) and higher scores on the ADAMS social avoidance subscale (b = .77 [95% CI: .41, 1.04], β = .76, p = .001).

### Prader–Willi Syndrome

For PWS, none of the overall models were significant and the addition of the predictors in Step 2 accounted for an additional 11–28% of the variance. For the individual predictors, higher adaptive ability was the only predictor of psychopathology and significantly predicted a lower total score (b = − 1.41 [95% CI: − 2.26, − .43], β = − .56, p = .001), lowered generalised anxiety (b = − .37 [95% CI: − .63, − .04], β = − .52, p = .006) and manic/hyperactivity (b = − .38 [95% CI: − .59, − .13], β = − .59, p = .003) on the ADAMS.

### Fragile X Syndrome

Only the overall model for the DBC mean item score for FXS was significant, (F(5,41) = 5.01, p = .001, R^2^ = .38). The addition of health and sensory processing in Step 2 accounted for an additional 3–20% of the variance across the outcome variables. The predictors for FXS differed from those for WS and PWS, with no associations identified for age, adaptive ability, health and auditory sensory processing.

With regard to overall level of psychopathology, auditory sensory processing impairments and a higher number of health problems were associated with general psychiatric problems in individuals with WS; poorer adaptive ability was associated with psychopathology for individuals with PWS. No associations were identified between any of the individual predictors and psychopathology for individuals with FXS.

## Discussion

This study is the first to examine differences in internalising psychopathology and potential predictors of general psychopathology and specific symptoms in individuals with WS, FXS and PWS. These syndromes were chosen for comparison as they are broadly comparable for degree of ID. Exploratory analyses (p < .01) focused on associations with age, adaptive ability, health and auditory sensory processing. To examine the impact of methodological assessment on the results obtained, both dimensional and categorical measures were used. The study identified cross-syndrome differences in: (1) health and auditory sensory processing, (2) psychopathology generally and (3) correlates of psychopathology (both general psychopathology and specific symptoms).

Profiles of psychopathology and associated factors differed across groups. Higher rates of auditory sensory processing impairments were found for individuals with WS and FXS compared with PWS; health difficulties were more prevalent in WS and PWS compared with FXS (small effect sizes). Consistent with our hypotheses and past research, generalised anxiety was significantly higher in FXS and WS compared with PWS, and both ADAMS and DBC social subscales were elevated in FXS compared with WS and PWS, although effect sizes were small. Our hypothesis that individuals with PWS would score higher on the compulsive behaviour subscale of the ADAMS was not supported. The high proportion of adults in this study (80% aged over 18) may account for this non-significant finding, as there is some evidence that compulsive behaviour decreases with age in PWS (Dykens 2004).

Cross-syndrome comparisons of correlates of psychopathology were also examined. Age was not predictive of psychopathology for any of the syndrome groups, consistent with several studies in the literature (Dykens and Kasari 1997; Hessl et al. 2001; Woodruff-Borden et al. 2010). However, as noted above, participants in this study were predominantly adults and thus the somewhat restricted age range, together with small samples sizes, may have reduced correlations with age. Larger samples including a broader age range are needed to provide further information on this relationship.

### Associations with Adaptive and Intellectual Functioning

Adaptive functioning did not predict psychopathology for FXS or WS, however poorer adaptive functioning was associated with total psychopathology, general anxiety and manic/hyperactive behaviour in individuals with PWS. PWS is caused by three genetic mechanisms, deletion, maternal uniparental disomy and imprinting defects (Cassidy et al. 2011). The deletion subtype has been associated with poorer adaptive functioning than the other subtypes (Butler et al. 2004) and since half of the sample in the study presented with the deletion subtype, this may have inflated the findings. However, despite this potential confound, the majority of research suggests limited differentiation in clinical features between the different PWS subtypes (Cassidy et al. 2011).

The findings in this study have general implications for identifying the underlying causes of psychopathology for WS, PWS and FXS. Some psychiatric difficulties in PWS seem to be associated with adaptive skills, whereas psychopathology in FXS and WS seems to be independent of ID or adaptive functioning and may be more phenotypically driven. This is generally consistent with existing literature suggesting that intellectual functioning is unrelated to the development of psychopathology in WS and FXS (Dodd and Porter 2009; Cordeiro et al. 2011; Stinton et al. 2010; Vasa et al. 2013). Whilst adaptive functioning has received limited attention, this study confirms a lack of association between adaptive ability and psychopathology in these two groups, consistent with previous cognitive and mental health research. In PWS, future research with larger samples and the consideration of each genetic subtype individually may further elucidate the association between psychopathology and adaptive functioning.

### Associations with Health Problems and Auditory Sensory Processing

This study is the first to identify an association between health and psychopathology in WS. Individuals with WS reported the highest number of health problems and current health difficulties were predictive of level and intensity of emotional disturbance, as measured by the DBC. Auditory sensory processing impairments were also only predictive of psychopathology for the WS group and were not predictive in individuals with FXS, despite these groups scoring at similar levels on the auditory subscale of the SEQ.

These findings suggest that difficulties with auditory sensory processing, such as the presence of hyperacusis, could be a specific risk marker for general psychopathology in individuals with WS. Hyperacusis is reported to affect up to 80% of individuals with WS (Levitin et al. 2005), hence this may partly contribute to the high rates of psychopathology reported in this syndrome. An association between social avoidance and sensory processing was also identified in participants with WS. Social anxiety has been associated with sensory processing sensitivities in clinical samples of individuals with social anxiety disorder and has also been identified in individuals with autism spectrum condition (Hilton et al. 2007; Hofmann and Bitran 2007). Future research is needed to investigate the relationship between sensory processing, social impairments and anxiety in WS and to explore how, and why, sensory processing problems appear to predispose to psychopathology in WS but not FXS.

It is notable that associations between psychopathology and health and sensory processing were identified in WS but not in the other two syndromes, contrary to expectation and despite a comparably high prevalence of health difficulties in PWS and auditory sensory impairments in FXS. The lack of significant associations between these variables in FXS and PWS suggests that these factors are not strongly implicated in the development of mental health problems for these groups. Instead, alternative predictors, not examined in this study, may play an influential role, such as the presence of autism (Hatton et al. 2002). These findings also suggest the presence of these characteristics alone are insufficient to influence psychopathological development and there may be additional biological processes in individuals with WS that interact with these variables to increase susceptibility to psychopathology. This, coupled with increased exposure to aversive health and sensory related experiences, may increase the risk for developing psychopathology in people with WS.

It is evident that the disparities in mental health correlates between genetic syndrome requires further extensive investigation. The identification of specific correlates has important implications for the understanding of causal pathways and the influence of risk factors for psychopathology in these groups. Further research in this area will also be essential to inform early intervention, which should be targeted towards addressing these predictors to reduce the onset and severity of subsequent mental health problems.

### Assessment Measures

The study demonstrates the importance of consideration of the measures used to assess psychopathology in genetic syndromes. The association between psychopathology and adaptive behaviour in PWS was only identified on the ADAMS subscales; no relationships were found on the DBC. Similarly, in WS, current health problems were predictive of psychopathology as measured by the DBC, but not when measured by the ADAMS. This indicates that results from studies using single approaches should be interpreted with caution and the influence of specific assessment methodologies on research findings should always be carefully considered. These findings also highlight the necessity for the use of multi-assessment approaches when examining psychopathology and potential correlates or risk markers.

### Limitations

There are several limitations of the study that should be taken into consideration. Firstly, assessments of psychopathology were based on questionnaire data, not clinical diagnosis. Whilst this methodology was chosen to provide continuous scores of the main topographies of psychopathology, and provides an indication of whether individuals may be experiencing clinically relevant disorders, it is neither as detailed nor robust as a clinical diagnostic assessment. Moreover, the sample sizes were uneven between the groups and may be considered small for the regression analyses conducted. However, according to Austin and Steyerberg (2015), two participants per variable is adequate to estimate regression coefficients. Under these guidelines and the through the use of bootstrapping, the sample sizes were considered adequate for the use of this analysis, although larger sample sizes are always preferred to ensure a greater level of statistical power. Larger samples may also help to overcome the small effect sizes identified for the significant results found in this study. Due to the exploratory nature of the study, the p-value chosen increases the probability of a type I error, hence results should be interpreted with this consideration.

In addition, this study utilised cross-sectional methodology whereas longitudinal research is needed to demonstrate the associations between predictors and mental health difficulties over time. The study also utilised a sample with a wide age range, however psychopathology may follow a developmental trajectory in these groups and the inclusion of children and adults in the sample does not allow for the exploration of age specific outcomes. Even so, age was included as a potential confound in Step 1 of the regression analysis and did not produce any significant results. Other studies examining the effects of age for these syndromes have also found limited results (Dykens and Kasari 1997; Hessl et al. 2001; Stinton et al. 2010), suggesting this may not be a significant concern. Finally, any voluntary questionnaire studies of this kind are prone to substantial bias (Keiding and Louis 2016); it is not known what proportion of families who had access to the surveys did respond, or how data from non-responders might have affected the findings.

Despite these caveats, we identified significant and potentially important cross-syndrome differences in the correlates of psychopathology for individuals with WS, PWS and FXS. Current health problems and auditory sensory processing deficits were associated with psychiatric disturbance for individuals with WS, and poorer adaptive functioning was predictive of higher psychopathology for individuals with PWS. None of the variables examined was predictive of psychopathology in FXS. This study highlights the need for continued research into risk markers, utilising a combination of approaches and the development of targeted syndrome-specific interventions to reduce and manage psychopathological disorders.

## Electronic supplementary material

Below is the link to the electronic supplementary material.

Supplementary material 1 (PDF 384 kb)
