# Supplementary material for: The Profiles and Correlates of Psychopathology in Adolescents and Adults with Williams, Fragile X and Prader–Willi Syndromes
Source: J Autism Dev Disord. 2019 Dec 4;50(3):893–903. doi: 10.1007/s10803-019-04317-1 (PMC7010621; doi:10.1007/s10803-019-04317-1)
Supplement: Supplementary file 1 — Supplementary material 1 (PDF 384 kb) [file 10803_2019_4317_MOESM1_ESM.pdf]

THE PROFILES AND CORRELATES OF PSYCHOPATHOLOGY IN ADOLESCENTS AND ADULTS WITH WILLIAMS, FRAGILE X AND PRADER-WILLI SYNDROMES

(withheld for blinded review)

Journal of Autism and Developmental Disorders

Supplemental Material

Online Resource A

Supplemental Table 1 Correlations between the ADAMS subscales for Williams syndrome, Fragile X syndrome and Prader-Willi syndrome

	Depressed Mood	General Anxiety	Manic/ hyperactivity	Obsessive-compulsive	Social Avoidance	ADAMS total
**Williams syndrome**						
Depressed mood	-	.71**	.61**	.40	.47*	.81**
General anxiety		-	.76**	.51*	.57**	.89**
Manic/hyperactivity			-	.49*	.66**	.85**
Obsessive-compulsive				-	.69**	.69**
Social Avoidance					-	.78**
ADAMS total						-
**Fragile X syndrome**						
Depressed mood	-	.72**	.53**	.39*	.28	.71**
General anxiety		-	.63**	.54**	.41*	.86**
Manic/hyperactivity			-	.59**	.52**	.84**
Obsessive-compulsive				-	.32	.72**
Social Avoidance					-	.68**
ADAMS total						-
**Prader-Willi syndrome**						
Depressed mood	-	.88**	.69**	.67**	.67**	.94**
General anxiety		-	.79**	.66**	.72**	.95**
Manic/hyperactivity			-	.59*	.51*	.82**
Obsessive-compulsive				-	.47	.74**
Social Avoidance					-	.77**
ADAMS total						-

*p<.01, **p<.0001

Online Resource B

Supplemental Table 2 Spearman's rho correlations between the ADAMS subscales and DBC mean item and intensity scores (n=110)

	**ADAMS subscales**					
	Depressed mood	Generalised anxiety	Manic/ hyperactive	Obsessive compulsive	Social avoidance	Total
**DBC score**						
Mean item score	.53**	.67**	.75**	.64**	.51**	.80**
Intensity score	.37**	.53**	.65**	.60**	.46**	.67**

**p<.0001

Online Resource C

Supplemental Table 3 Group differences between Williams syndrome (n=35), fragile X syndrome (n=49) and Prader-willi syndrome (n=26) for age, gender, level of independence, auditory sensory processing, health and emotional/behavioural measures

		**WS**	**FXS**	**PWS**	F/x^2	P value <.01	ε^2	Post hoc tests <.01
**Age**	Med (IR)<br>range	21.0 (45)<br>12-57	24.0 (12)<br>12-50	25.5 (15)<br>12-47	1.93	.381		
**Gender**	% of males	40	100	42	43.02	<.0001	.63[a]	FXS>PWS, WS
**WADL** *total score*	M (SD)<br>range	18.37 (6.24)<br>6-28	18.87 (5.45)<br>9-29	17.50 (5.84)<br>4-29	.47	.626		
**SEQ** *auditory score*		18.69 (3.36)<br>13-25	17.64 (3.88)<br>10-26	13.19 (3.12)<br>8-20	19.67	<.0001	.27[b]	FXS, WS> PWS
**Health**								
Lifetime overall scores	Med (IR)<br>range	9.0 (4.0)<br>2-22	5.0 (3.0)<br>0-16	6.0 (8.0)<br>1-23	23.90	<.0001	.22	WS>FXS
Lifetime no. of difficulties		6.0 (2.0)<br>2-11	3.0 (3.0)<br>0-8	5.0 (4.0)<br>1-10	27.97	<.0001	.26	PWS, WS >FXS
Current overall scores		3.0 (5.0)<br>0-12	0.0 (2.0)<br>0-16	2.0 (3.3)<br>0-19	15.15	.001	.14	PWS, WS >FXS
Current no. of difficulties		2.0 (3.0)<br>0-8	0.0 (1.0)<br>0-8	1.0 (2.0)<br>0-10	18.74	<.0001	.17	PWS, WS>FXS

[a] Cramer's V for effect size

[b] Partial eta squared (η_p^2) for effect size

	**WS**<br>**Med (IR)**<br>**range**	**FXS**<br>**Med (IR)**<br>**range**	**PWS**<br>**Med (IR)**<br>**range**	F/x^2	P value<br><.01	ε²	Post hoc tests<br><.05
**ADAMS**							
Depressed mood	4.0 (7.0)<br>0-21	2.0 (5.0)<br>0-14	5.0 (7.0)<br>0-18	6.49	.039		
General anxiety	7.0 (6.0)<br>0-21	8.0 (7.0)<br>0-20	4.0 (8.0)<br>0-12	9.61	.008	.09	FXS, WS >PWS
Manic hyperactive	4.0 (7.0)<br>0-12	7.0 (7.0)<br>0-14	3.0 (6.5)<br>0-12	10.16	.006	.09	FXS> PWS
Obsessive compulsive	1.0 (3.0)<br>0-9	2.0 (5.0)<br>0-9	3.0 (4.0)<br>0-6	1.60	.450		
Social avoidance	3.0 (5.0)<br>0-16	8.0 (7.0)<br>2-17	2.0 (5.3)<br>0-12	37.13	<.0001	.34	FXS>PWS, WS
Total	18.0 (23.0)<br>1-70	25.0 (21.0)<br>4-66	18.0 (27.5)<br>0-45	6.61	.037		
**DBC A**							
Antisocial	4.0 (5.0)<br>0-24	5.0 (5.0)<br>1-12	9.0 (11.0)<br>1-22	6.46	.040		
Self-absorbed	6.0 (8.0)<br>0-33	8.0 (4.0)<br>2-23	6.0 (8.0)<br>1-28	3.54	.170		
Communication and anxiety	11.0 (10.0)<br>1-29	15.0 (7.75)<br>6-36	13.0 (12.5)<br>3-30	3.99	.136		
Disruptive	9.0 (11.5)<br>1-41	10.0 (9.50)<br>2-33	16.0 (14.5)<br>2-32	2.93	.231		
Social relating	3.0 (4.0)<br>0-13	8.0 (4.0)<br>3-14	4.0 (4.0)<br>0-12	26.96	<.0001	.32	FXS>PWS, WS
Depressive	5.0 (8.0)<br>0-19	2.0 (4.0)<br>0-20	6.0 (8.0)<br>0-14	5.82	.054		
Total	38.0 (36.0)<br>5-129	45.5 (24.3)<br>21-107	47.0 (46.5)<br>12-117	1.86	.395		

	WS Med (IR) range	FXS Med (IR) range	PWS Med (IR) range	F/x^2	P value <.01	ε^2	Post hoc tests <.05
**DBC P**[c]							
Disruptive/ antisocial	14.5 (19.5) 2-32	30.0 (23.8) 8-40	10.0 (22.5) 3-31				
Self-absorbed	9.0 (15.0) 2-36	31.5 (29.8) 7-45	7.0 (25.5) 2-42				
Communication	8.5 (5.8) 2-17	17.5 (18.3) 3-25	8.0 (10.0) 1-16				
Anxiety	5.0 (5.0) 0-10	6.0 (5.0) 4-12	3.0 (3.0) 1-5				
Social relating	3.5 (5.5) 0-13	7.5 (7.3) 4-12	5.0 (3.0) 0-5				
Total	45.0 (45.0) 11-107	96.5 (83.0) 31-126	35.0 (60.5) 8-101				
**DBC Combined scores**							
Mean item score	.36 (.40) .05-1.21	.45 (.29) .06-1.31	.43 (.42) .08-1.09	2.40	.301		
Positively checked items	.18 (.20) .02-.60	.22 (.15) .03-.66	.21 (.21) .04-.55	2.39	.303		
Intensity score	.06 (.14) .00-.50	.10 (.12) .00-.60	.08 (.20) .00-.40	1.90	.387		
**Anxiety triggers**							
Total score	46.0 (31.3) 11-87	44.4 (30.3) 7-93	20.5 (30.0) 0-64	18.42	<.0001	.17	FXS, WS> PWS
**Setting events %**							
Tiredness	50	44	69	4.41	3.49		
Pain/illness	65	45	46	3.49	.174		
Hunger	41	46	85	13.21	.001	.14	PWS> FXS, WS
Negative Mood	59	52	62	.72	.699		

Online Resource D

Regression linear models of predictors of mental health difficulties for Williams syndrome, Fragile X syndrome and Prader-Willi syndrome.

Williams syndrome

Supplemental Table 4 **WS - Total Anxiety, Depression and Mood score** Linear model of predictors of total anxiety, depression and mood score, with 95% bias corrected and accelerated confidence intervals reported in parentheses. Confidence intervals and standard errors based on 1000 bootstrap samples.

	b	SE B	β	p
Step 1				
Constant	41.97<br>(21.05, 70.96)	12.20		**.001**
Age	-100.10<br>(-361.33, 174.39)	137.63	-.13	.449
Adaptive ability	-0.76<br>(-1.60, -0.11)	0.45	-.28	.100
Step 2				
Constant	-24.08<br>(-71.33, 71.20)	23.85		.220
Age	-161.72<br>(-383.62, 26.85)	101.86	-.20	.101
Adaptive ability	0.03<br>(-0.78, 0.55)	0.49	.01	.948
Lifetime health	0.29<br>(-0.78, 1.67)	0.55	.08	.544
Current health	2.50<br>(0.22, 4.51)	0.94	.50	.014
Auditory processing	2.31<br>(0.83, 3.41)	0.96	.46	.012

Supplemental Table 5 **WS - Depressed mood** Linear model of predictors of depressed mood, with 95% bias corrected and accelerated confidence intervals reported in parentheses. Confidence intervals and standard errors based on 1000 bootstrap samples.

	b	SE B	β	p
Step 1				
Constant	10.55<br>(3.46, 19.75)	4.01		.018
Age	-50.87<br>(-14.71, 44.20)	46.17	-.22	.279
Adaptive ability	-0.16<br>(-0.43, 0.04)	0.12	-.21	.199
Step 2				
Constant	.063<br>(-17.16, 22.92)	7.76		.993
Age	-53.31<br>(-132.80, 14.49)	32.77	-.23	.106
Adaptive ability	-0.09<br>(-0.35, 0.11)	0.15	-.12	.492
Lifetime health	0.22<br>(-0.14, 0.63)	0.17	.21	.169
Current health	0.72<br>(0.05, 1.14)	0.28	.50	.018
Auditory processing	0.25<br>(-0.32, 0.72)	0.30	.18	.367

Supplemental Table 6 **WS - General anxiety** Linear model of predictors of generalised anxiety, with 95% bias corrected and accelerated confidence intervals reported in parentheses. Confidence intervals and standard errors based on 1000 bootstrap samples.

	b	SE B	β	p
Step 1				
Constant	12.99<br>(6.62, 21.48)	3.29		**.002**
Age	-31.22<br>(-107.23, 58.61)	42.65	-.13	.204
Adaptive ability	-0.19<br>(-0.43, 0.01)	0.13	-.23	.479
Step 2				
Constant	-4.16<br>(-16.74, 21.32)	8.00		.596
Age	-36.68<br>(-101.43, 30.22)	34.63	-.15	.869
Adaptive ability	-0.03<br>(-0.43, 0.01)	0.17	-.03	.323
Lifetime health	0.28<br>(-0.10, 0.67)	-.20	.25	.141
Current health	0.59<br>(-0.12, 1.22)	0.31	.39	.026
Auditory processing	0.52<br>(-0.08, 0.87)	0.30	.34	.085

Supplemental Table 7 **WS - Manic/Hyperactivity** Linear model of predictors of manic/hyperactivity, with 95% bias corrected and accelerated confidence intervals reported in parentheses. Confidence intervals and standard errors based on 1000 bootstrap samples.

	b	SE B	β	p
Step 1				
Constant	7.43<br>(0.69, 15.04)	3.77		.016
Age	12.29<br>(-60.11, 92.21)	38.99	.07	.706
Adaptive ability	-0.17<br>(-0.46, 0.08)	0.13	-.28	.123
Step 2				
Constant	-6.83<br>(-19.03, 16.65)	8.09		.273
Age	7.52<br>(-47.32, 53.79)	32.20	.04	.795
Adaptive ability	-0.02<br>(-0.36, 0.17)	0.16	-.04	.850
Lifetime health	0.21<br>(-0.02, 0.44)	0.14	.26	.153
Current health	0.31<br>(0.22, 0.77)	0.22	.27	.132
Auditory processing	0.47<br>(-0.07, 0.76)	0.27	.41	.052

Supplemental Table 8 **WS - Obsessive Compulsive** Linear model of predictors of obsessive compulsive, with 95% bias corrected and accelerated confidence intervals reported in parentheses. Confidence intervals and standard errors based on 1000 bootstrap samples.

	b	SE B	β	p
Step 1				
Constant	4.32<br>(1.28, 8.40)	1.78		.021
Age	-22.11<br>(-59.92, 16.69)	19.47	-.19	.226
Adaptive ability	-0.06<br>(-0.19, 0.05)	0.07	-.16	.390
Step 2				
Constant	-0.44<br>(-9.55, 9.66)	3.94		.898
Age	-37.33<br>(-79.04, -1.36)	20.80	-.32	.069
Adaptive ability	0.03<br>(-0.13, -0.17)	0.09	.07	.725
Lifetime health	-0.18<br>(-0.38, 0.07)	0.10	-.33	.095
Current health	0.35<br>(-0.04, 0.61)	0.16	.47	.041
Auditory processing	0.23<br>(-0.07, 0.50)	0.16	.31	.148

Supplemental Table 9 **WS - Social Avoidance** Linear model of predictors of social avoidance, with 95% bias corrected and accelerated confidence intervals reported in parentheses. Confidence intervals and standard errors based on 1000 bootstrap samples.

	b	SE B	β	p
Step 1				
Constant	6.15<br>(2.54, 10.58)	1.99		**.010**
Age	-5.31<br>(-50.70, 52.36)	26.67	-.03	.838
Adaptive ability	-0.15<br>(-0.30, -0.03)	-0.08	-.29	.057
Step 2				
Constant	-12.42<br>(-25. 19, 2.98)	5.04		.012
Age	-30.78<br>(-78.15, 16.35)	22.35	-.19	.170
Adaptive ability	-0.11<br>(-0.06, 0.25)	0.10	.21	.261
Lifetime health	0.42<br>(-0.33, 0.24)	0.13	-.12	.515
Current health	-0.09<br>(-0.08, 0.76)	0.24	.42	.077
Auditory processing	0.77<br>(0.41, 1.04)	0.20	.76	**.001**

Supplemental Table 10 WS - DBC MIS Linear model of predictors of DBC MIS, with 95% bias corrected and accelerated confidence intervals reported in parentheses. Confidence intervals and standard errors based on 1000 bootstrap samples.

	b	SE B	β	p
Step 1				
Constant	0.62<br>(0.14, 1.19)	0.24		**.008**
Age	2.19<br>(-2.10, 7.25)	2.43	.16	.368
Adaptive ability	-0.02<br>(-0.03, -0.00)	0.01	-.32	.084
Step 2				
Constant	-0.63<br>(-1.39, 1.05)	0.44		.076
Age	1.01<br>(-2.24, 3.99)	1.60	.07	.494
Adaptive ability	0.00<br>(-0.01, 0.01)	0.01	.04	.786
Lifetime health	0.00<br>(-0.02, 0.02)	0.01	-.01	.961
Current health	0.05<br>(0.02, 0.09)	0.01	.52	**.001**
Auditory processing	0.04<br>(0.01, 0.06)	0.02	.50	**.008**

Supplemental Table 11 **WS - DBC II** Linear model of predictors of DBC II, with 95% bias corrected and accelerated confidence intervals reported in parentheses. Confidence intervals and standard errors based on 1000 bootstrap samples.

	b	SE B	β	p
Step 1				
Constant	0.18<br>(0.01, 0.41)	0.11		.094
Age	1.06<br>(-0.71, 3.27)	0.99	.17	.275
Adaptive ability	-0.01<br>(-0.02, 0.00)	0.00	-.28	.108
Step 2				
Constant	-0.31<br>(-0.73, 0.29)	0.19		.064
Age	0.29<br>(-1.49, 2.15)	0.79	.05	.678
Adaptive ability	0.00<br>(-0.01, 0.01)	0.00	.08	.624
Lifetime health	-0.01<br>(-0.02, 0.00)	0.01	-.22	.149
Current health	0.02<br>(0.01, 0.04)	0.01	.55	**.009**
Auditory processing	0.02<br>(0.01, 0.03)	0.01	.49	.013

Fragile X syndrome

Supplemental Table 12 **FXS - Total Anxiety, Depression and Mood score** Linear model of predictors of total anxiety, depression and mood score, with 95% bias corrected and accelerated confidence intervals reported in parentheses. Confidence intervals and standard errors based on 1000 bootstrap samples.

	b	SE B	β	p
Step 1				
Constant	35.79 (13.92-59.46)	11.48		**.003**
Age	-32.29 (-334.23-267.17)	142.93	-.04	.816
Adaptive ability	-0.36 (-1.16-0.41)	0.40	-.14	.370
Step 2				
Constant	12.85 (-23.52-56.37)	16.09		.368
Age	-37.07 (-300.80-154.88)	147.04	-.04	.793
Adaptive ability	-0.02 (-0.82-0.66)	0.44	.01	.969
Lifetime health	0.83 (-0.89-2.45)	0.83	.20	.329
Current health	0.67 (-1.46-5.70)	1.34	.13	.524
Auditory processing	0.67 (-0.43-1.59)	0.57	.17	.230

Supplemental Table 13 **FXS - Depressed Mood** Linear model of predictors of depressed mood score, with 95% bias corrected and accelerated confidence intervals reported in parentheses. Confidence intervals and standard errors based on 1000 bootstrap samples.

	B (95% CIs)	SE B	β	p
Step 1				
Constant	2.11 (-2.44-6.31)	2.26		.347
Age	-3.93 (-52.35-60.12)	27.55	-.02	.873
Adaptive ability	0.04 (-0.10-0.19)	0.07	.07	.607
Step 2				
Constant	-2.62 (-13.19-6.22)	4.06		.531
Age	-2.41 (-61.02-46.31)	33.97	-.01	.932
Adaptive ability	0.12 (-0.01-0.26)	0.09	.22	.178
Lifetime health	0.13 (-0.23-0.53)	0.17	.16	.411
Current health	0.25 (-0.20-1.38)	0.35	.23	.346
Auditory processing	0.12 (-0.16-0.39)	0.15	.15	.442

Supplemental Table 14 **FXS - General anxiety** Linear model of predictors of generalised anxiety score, with 95% bias corrected and accelerated confidence intervals reported in parentheses. Confidence intervals and standard errors based on 1000 bootstrap samples.

	B (95% CIs)	SE B	β	p
Step 1				
Constant	7.18 (-0.41-14.26)	3.93		.061
Age	14.45 (-71.14-105.62)	45.06	.05	.718
Adaptive ability	-0.01 (-0.28-0.29)	0.14	-.02	.917
Step 2				
Constant	-2.00 (-14.06-9.86)	5.22		.682
Age	12.60 (-60.81-72.64)	45.72	.04	.797
Adaptive ability	0.10 (-0.15-0.37)	0.14	.12	.466
Lifetime health	0.30 (-0.27-0.83)	0.27	.23	.275
Current health	0.08 (-0.69-1.63)	0.46	.05	.815
Auditory processing	0.31 (-0.03-0.65)	0.18	.24	.090

Supplemental Table 15 **FXS - Manic/Hyperactivity** Linear model of predictors of manic hyperactive score, with 95% bias corrected and accelerated confidence intervals reported in parentheses. Confidence intervals and standard errors based on 1000 bootstrap samples.

	B (95% CIs)	SE B	β	p
Step 1				
Constant	9.00 (2.94-15.61)	2.97		**.002**
Age	23.43 (-66.63-100.40)	42.87	.09	.584
Adaptive ability	-0.16 (-0.37-0.02)	0.10	-.22	.111
Step 2				
Constant	5.45 (-6.26-16.57)	4.54		.219
Age	23.58 (-61.19-81.29)	45.65	.09	.594
Adaptive ability	-0.10 (-0.31-0.11)	0.12	-.13	.401
Lifetime health	0.12 (-0.31, 0.50)	0.21	.11	.562
Current health	0.28 (-0.32-1.41)	0.34	.19	.291
Auditory processing	0.07 (-0.20-0.33)	0.15	.07	.607

Supplemental Table 16 **FXS - Obsessive Compulsive** Linear model of predictors of obsessive compulsive score, with 95% bias corrected and accelerated confidence intervals reported in parentheses. Confidence intervals and standard errors based on 1000 bootstrap samples.

	B (95% CIs)	SE B	β	p
Step 1				
Constant	2.74 (-2.81-7.69)	2.57		.271
Age	31.39 (-33.13-103.69)	33.57	.16	.341
Adaptive ability	-0.06 (-0.21-0.11)	0.08	-.11	.470
Step 2				
Constant	-2.72 (-11.59-6.98)	3.60		.426
Age	28.80 (-32.57-71.99)	33.31	.15	.403
Adaptive ability	0.02 (-0.15-0.18)	0.09	.03	.855
Lifetime health	0.24 (-0.06-0.59)	0.15	.28	.110
Current health	0.10 (-0.33-0.93)	0.26	.09	.645
Auditory processing	0.16 (-0.06-0.34)	0.12	.20	.192

Supplemental Table 17 **FXS - Social Avoidance** Linear model of predictors of social avoidance score, with 95% bias corrected and accelerated confidence intervals reported in parentheses. Confidence intervals and standard errors based on 1000 bootstrap samples.

	B (95% CIs)	SE B	β	p
Step 1				
Constant	13.06 (5.81-20.73)	3.84		**.003**
Age	-69.93 (-157.91-24.38)	46.36	-.26	.131
Adaptive ability	-0.09 (-0.34-0.13)	0.12	-.12	.499
Step 2				
Constant	10.21 (-0.45-23.10)	5.70		.070
Age	-71.61 (-167.08-22.22)	48.99	-.27	.141
Adaptive ability	-0.05 (-0.33-0.21)	0.14	-.06	.759
Lifetime health	0.14 (-0.32-0.64)	0.23	.12	.550
Current health	0.07 (-0.52-0.83)	0.33	.05	.770
Auditory processing	0.08 (-0.35-0.40)	0.19	.07	.692

Supplemental Table 18 **FXS - DBC MIS** Linear model of predictors of DBC MIS score, with 95% bias corrected and accelerated confidence intervals reported in parentheses. Confidence intervals and standard errors based on 1000 bootstrap samples.

	B (95% CIs)	SE B	β	p
Step 1				
Constant	0.25 (-0.11-0.65)	0.20		.211
Age	6.92 (0.89-12.62)	3.18	.41	.037
Adaptive ability	-0.00 (-0.01-0.01)	0.01	-.04	.771
Step 2				
Constant	-0.04 (-0.64-0.74)	0.29		.887
Age	4.49 (-0.94-8.82)	2.92	.26	.137
Adaptive ability	0.00 (-0.01-0.01)	0.01	.03	.826
Lifetime health	0.03 (-0.01-0.05)	0.02	.30	.087
Current health	0.03 (-0.03-0.08)	0.03	.23	.203
Auditory processing	0.01 (-0.01-0.02)	0.01	.14	.273

Supplemental Table 19 **FXS - DBC II** Linear model of predictors of DBC II score, with 95% bias corrected and accelerated confidence intervals reported in parentheses. Confidence intervals and standard errors based on 1000 bootstrap samples.

	B (95% CIs)	SE B	β	p
Step 1				
Constant	0.06 (-0.12-0.25)	0.10		.534
Age	2.08 (-0.84-5.23)	1.18	.29	.183
Adaptive ability	-0.00 (-0.01-0.00)	0.00	-.05	.730
Step 2				
Constant	-0.08 (-0.37-0.25)	0.14		.559
Age	1.43 (-1.36-3.84)	1.19	.20	.319
Adaptive ability	0.00 (-0.01, 0.01)	0.00	.02	.898
Lifetime health	0.01 (-0.01, 0.02)	0.01	.16	.302
Current health	0.02 (-0.01-0.04)	0.01	.24	.224
Auditory processing	0.01 (-0.00, 0.01)	0.00	.18	.212

Prader-Willi syndrome

Supplemental Table 20 **PWS - Total Anxiety, Depression and Mood score** Linear model of predictors of total anxiety, depression and mood score, with 95% bias corrected and accelerated confidence intervals reported in parentheses. Confidence intervals and standard errors based on 1000 bootstrap samples.

	b	SE B	β	p
Step 1				
Constant	52.47<br>(28.56, 74.84)	12.96		**.001**
Age	-205.17<br>(-500.87, 187.12)	177.60	-.23	.233
Adaptive ability	-1.41<br>(-2.26, -0.43)	0.44	-.56	**.001**
Step 2				
Constant	26.34<br>(-28.50, 77.45)	26.78		.358
Age	-165.21<br>(-580.73, 335.01)	195.64	-.18	.373
Adaptive ability	-1.17<br>(-2.28, -0.02)	0.59	-.47	.052
Lifetime health	0.67<br>(-1.05, 2.28)	0.88	.26	.433
Current health	-0.33<br>(-2.32, 2.00)	0.94	-.12	.637
Auditory processing	1.23<br>(-0.93, 3.56)	1.38	.26	.377

Supplemental Table 21 **PWS - Depressed mood** Linear model of predictors of depressed mood, with 95% bias corrected and accelerated confidence intervals reported in parentheses. Confidence intervals and standard errors based on 1000 bootstrap samples.

	b	SE B	β	p
Step 1				
Constant	15.29<br>(8.74, 21.64)	3.10		**.001**
Age	-96.68<br>(-170.33, -16.65)	41.34	-.41	.014
Adaptive ability	-0.36<br>(-0.57, -0.08)	0.12	-.54	.011
Step 2				
Constant	1.00<br>(-5.40, 23.97)	0.24		.242
Age	-92.00<br>(-245.07, 60.67)	0.09	-.39	.085
Adaptive ability	-0.32<br>(-0.65, 0.05)	0.09	-.48	.086
Lifetime health	0.38<br>(-0.07, 0.75)	0.12	.55	.124
Current health	-0.25<br>(-0.78, 0.45)	0.23	-.33	.230
Auditory processing	0.18<br>(-0.40, 0.77)	0.64	.14	.637

Supplemental Table 22 **PWS - General anxiety** Linear model of predictors of generalised anxiety, with 95% bias corrected and accelerated confidence intervals reported in parentheses. Confidence intervals and standard errors based on 1000 bootstrap samples.

	b	SE B	β	p
Step 1				
Constant	12.04 (4.28, 19.17)	4.19		.011
Age	-28.41 (-130.98, 79.43)	54.42	-.11	.588
Adaptive ability	-0.37 (-0.63, -0.04)	0.14	-.52	**.006**
Step 2				
Constant	8.21 (-9.04, 22.62)	8.85		.406
Age	-29.07 (-170.01, 131.60)	60.76	-.11	.649
Adaptive ability	-0.35 (-0.73, 0.08)	0.19	-.49	.100
Lifetime health	0.38 (-0.09, 0.76)	0.28	.52	.151
Current health	-0.30 (-0.98, 0.50)	0.28	-.37	.204
Auditory processing	0.13 (-0.50, 0.86)	0.40	.10	.745

Supplemental Table 23 **PWS - Manic Hyperactivity** Linear model of predictors of manic hyperactive, with 95% bias corrected and accelerated confidence intervals reported in parentheses. Confidence intervals and standard errors based on 1000 bootstrap samples.

	b	SE B	β	p
Step 1				
Constant	11.29<br>(4.90, 17.35)	3.63		**.007**
Age	-14.57<br>(-99.21, 101.16)	52.26	-.06	.773
Adaptive ability	-0.38<br>(-0.59, -0.13)	0.11	-.59	**.003**
Step 2				
Constant	6.79<br>(-8.41, 19.43)	6.91		.362
Age	-14.03<br>(-102.74, 113.01)	52.49	-.06	.792
Adaptive ability	-0.36<br>(-0.61, -0.08)	0.13	-.55	.029
Lifetime health	0.29<br>(-0.11, 0.63)	0.22	.44	.207
Current health	-0.25<br>(-0.74, 0.16)	0.24	-.34	.265
Auditory processing	0.21<br>(-0.33, 0.91)	0.34	.17	.537

Supplemental Table 24 **PWS - Obsessive compulsive** Linear model of predictors of obsessive compulsive, with 95% bias corrected and accelerated confidence intervals reported in parentheses. Confidence intervals and standard errors based on 1000 bootstrap samples.

	b	SE B	β	p
Step 1				
Constant	5.11<br>(-0.31, 10.89)	2.58		.067
Age	-9.19<br>(-65.96, 60.38)	32.52	-.07	.777
Adaptive ability	-0.13<br>(-0.30, 0.01)	0.08	-.36	.146
Step 2				
Constant	0.69<br>(-9.02, 7.99)	4.51		.886
Age	6.81<br>(-60.67, 97.11)	37.47	.05	.854
Adaptive ability	-0.05<br>(-0.26, 0.17)	0.11	-.15	.615
Lifetime health	-0.07<br>(-0.33, 0.29)	0.14	-.19	.595
Current health	0.19<br>(-0.24, 0.52)	0.16	.46	.161
Auditory processing	0.17<br>(-0.17, 0.55)	0.19	.26	.341

Supplemental Table 25 **PWS - Social Avoidance** Linear model of predictors of social avoidance, with 95% bias corrected and accelerated confidence intervals reported in parentheses. Confidence intervals and standard errors based on 1000 bootstrap samples.

	b	SE B	β	p
Step 1				
Constant	11.24	3.80		**.009**
	(3.41, 18.72)			
Age	-78.81	41.91	-.34	.058
	(-159.18, 20.77)			
Adaptive ability	-0.26	0.13	-.40	.059
	(-0.52, -0.01)			
Step 2				
Constant	4.01	6.49		.531
	(-8.07, 18.25)			
Age	-60.58	47.80	-.26	.186
	(-163.52, 68.22)			
Adaptive ability	-0.17	0.15	-.27	.240
	(-0.48, 0.14)			
Lifetime health	-0.19	0.28	-.29	.436
	(-0.73, 0.22)			
Current health	0.22	0.30	.30	.346
	(-0.29, 0.94)			
Auditory processing	0.43	0.32	.35	.175
	(-0.07, 0.87)			

Supplemental Table 26 **PWS - DBC MIS** Linear model of predictors of DBC MIS, with 95% bias corrected and accelerated confidence intervals reported in parentheses. Confidence intervals and standard errors based on 1000 bootstrap samples.

	b	SE B	β	p
Step 1				
Constant	0.90<br>(0.44, 1.31)	0.29		**.007**
Age	-0.53<br>(-8.57, 9.36)	4.37	-.03	.894
Adaptive ability	-0.02<br>(-0.04, 0.01)	0.01	-.45	.018
Step 2				
Constant	0.13<br>(-0.81, 0.84)	0.48		.800
Age	1.32<br>(-6.36, 11.74)	3.97	.08	.736
Adaptive ability	-0.01<br>(-0.04, 0.02)	0.01	-.25	.235
Lifetime health	0.01<br>(-0.02, 0.03)	0.02	.25	.414
Current health	0.01<br>(-0.03, 0.09)	0.02	.08	.739
Auditory processing	0.03<br>(-0.01, 0.08)	0.02	.34	.171

Supplemental Table 27 PWS - DBC II Linear model of predictors of DBC II, with 95% bias corrected and accelerated confidence intervals reported in parentheses. Confidence intervals and standard errors based on 1000 bootstrap samples.

	b	SE B	β	p
Step 1				
Constant	0.15	0.13		.227
	(-0.09, 0.34)			
Age	1.36	1.83	.20	.449
	(-1.37, 5.88)			
Adaptive ability	-0.01	0.01	-.27	.258
	(-0.01, 0.01)			
Step 2				
Constant	-0.10	0.16		.524
	(-0.41, 0.12)			
Age	2.01	1.71	.30	.229
	(-0.46, 5.96)			
Adaptive ability	-0.00	0.01	-.09	.742
	(-0.01, 0.01)			
Lifetime health	0.01	0.01	.24	.488
	(-0.01, 0.01)			
Current health	0.00	0.01	.09	.759
	(-0.01, 0.05)			
Auditory processing	0.01	0.01	.25	.229
	(-0.01, 0.03)			
